# Ultrasonographic Diagnosis of *Schistosoma mansoni* Eggs in Rectum

**DOI:** 10.1155/2016/5438204

**Published:** 2016-08-07

**Authors:** Fabio G. Rodrigues, Joao Batista Campos, Nivaldo Hartung Toppa, Steven D. Wexner, Giovanna Dasilva

**Affiliations:** ^1^Department of Colorectal Surgery, Cleveland Clinic Florida, Weston, FL 33331, USA; ^2^Endoscopia Clínica e Cirúrgica, 30150-270 Belo Horizonte, MG, Brazil; ^3^CNPq, 71605-001 Brasilia, DF, Brazil; ^4^Laboratório ANALYS Patologia, 30130-130 Belo Horizonte, MG, Brazil

## Abstract

Schistosomiasis is a trematode infection endemic in more than 70 countries that affects an estimated 250 million people. We report the case of a 60-year-old healthy female referred for endoscopic ultrasound after rectal examination revealed granular lesions. Ultrasound revealed the presence of deep mucosal nodular lesions with calcified/hyperechoic inclusions. Histologic evaluation has confirmed the final diagnosis of chronic schistosomal colitis. In patients with nonspecific intestinal lesions without a suspected diagnosis of schistosomiasis, endoscopic ultrasound can be enlightening. Schistosomiasis is still an endemic infection in some parts of Brazil and other tropical regions, causing colorectal lesions with unspecific presentation.

## 1. Introduction

Schistosomiasis is a trematode infection endemic in more than 70 countries that affects an estimated 250 million people [1]. The most commonly affected countries are in tropical and subtropical regions. The infection is caused by* Schistosoma *sp. and is transmitted by fresh water snails. After the cercaria penetrates the skin, the parasites migrate to the lungs and mature in the liver. The adult worms pair up in the hepatic portal vein and start to produce ova. There are five species of* Schistosoma* that cause schistosomiasis in humans and are distributed throughout specific geographic areas.* S. mansoni *is prevalent in Africa, the Middle East, and South America.* S. japonicum* is responsible for endemic areas in Asia.* S. haematobium* is present in Africa, the Middle East, and India.* S. intercalatum* occurs in Central America and West Africa, and* S. mekongi* can be found in Laos and Cambodia.

Since the 1970s, a few programs to control this epidemic were implemented in various regions. Some programs were successful, notably in Japan where the disease was eradicated in 1977 and in Brazil, where a hyperendemic area witnessed a dramatic decrease in the prevalence of schistosomiasis from 70.4% in 1981 to 1.7% in 2005 [2–4]. However, there are still endemic areas and in some regions the prevalence has actually increased after the implementation of control programs.

*S. mansoni*,* S. japonicum*,* S. intercalatum, *and* S. mekongi *can cause the gastrointestinal form of schistosomiasis [1]. Colonic pathologies have been described after infection because the parasitic eggs cause an inflammatory reaction. Since colonic disease may have a mild presentation or may even be asymptomatic, colorectal evaluation can be neglected. The most morbid event is hepatosplenic disease, which takes precedence in the treatment of these patients. We report the case of a patient referred for endoscopic ultrasound after rectal examination revealed granular lesions.

## 2. Case Report

**Figure 1:**
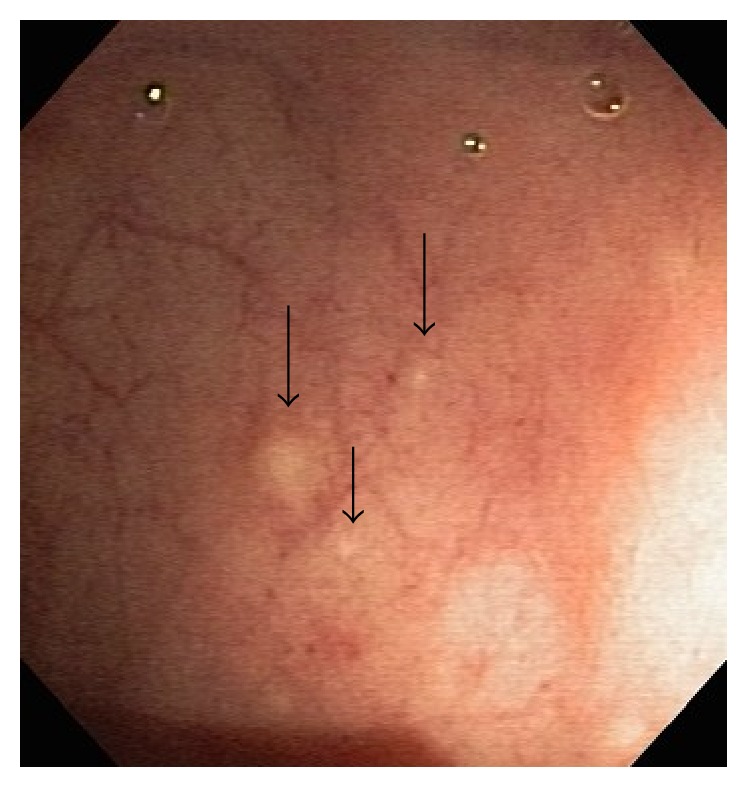
Colonoscopic view of yellowish subepithelial nodes in rectum (black arrows).

A 60-year-old healthy asymptomatic female underwent screening colonoscopy with findings of yellowish rectal subepithelial nodules (measuring up to 5 mm in diameter) (Figure 1). The patient was referred for endoscopic ultrasound. The patient lived in an endemic area for schistosomiasis in Brazil and had a positive history of freshwater bathing.

**Figure 2:**
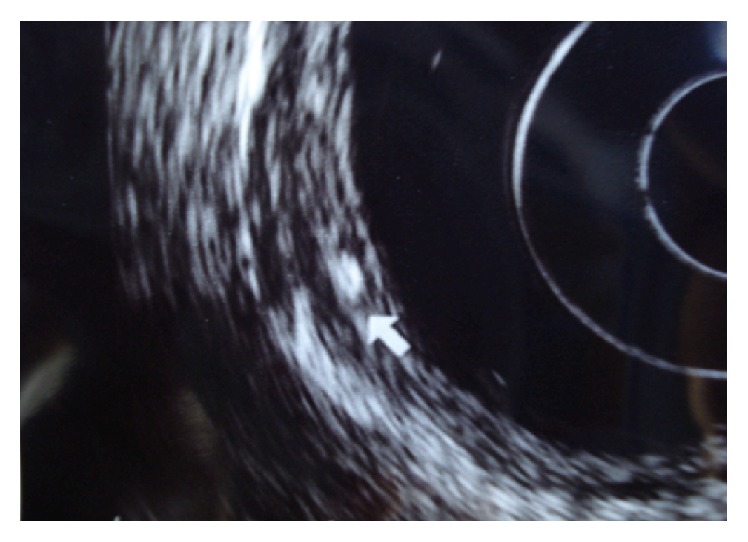
Endoscopic ultrasound image of a submucosal granuloma with calcified egg in its interior (white arrow).

Endoscopic examination was performed under sedation and monitored by an anesthesiologist. A multifrequency radial endoscope was used. Ultrasound revealed the presence of deep mucosal nodular lesions with calcified/hyperechoic inclusions (Figure 2). There were no alterations in the deeper colonic wall layers. A diagnosis of schistosomal granulomas was considered and biopsies were performed.

**Figure 3:**
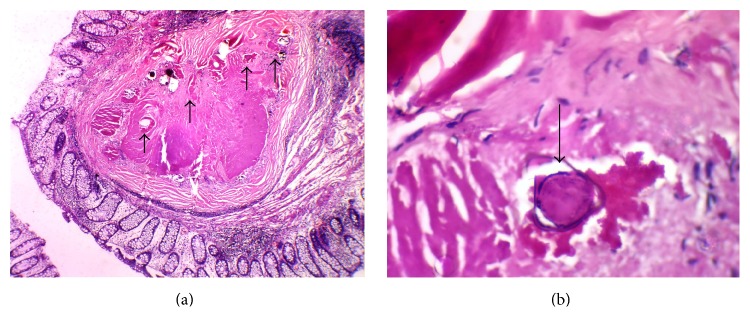
(a) Histological aspect—confluent granulomas with fibrosis, containing calcified* S. mansoni* eggs (black arrows) (HE 100x). (b) Histological aspect—detailed view of a* S. mansoni* partially calcified egg surrounded by hyaline fibrosis (black arrow) (HE 400x).

Histologic analysis revealed fragments of colonic mucosa showing increased cellularity in the* lamina propria*, with mononuclear inflammatory infiltrate and lymphoid follicles. In addition, granulomas in the healing phase were noted with hyaline fibrosis and calcified areas of* Schistosoma mansoni. *There were no viable eggs noted in the histologic evaluation. The final diagnosis was chronic schistosomal colitis (Figure 3). The patient was treated with praziquantel (40 mg/kg body weight).

## 3. Discussion

Involvement of the colon and rectum in schistosomiasis occurs as part of the natural cycle of the parasite. The two most important species associated with the gastrointestinal form of schistosomiasis are* S. mansoni and S. japonicum* [5]. The adult female worms travel against the blood stream, driven by oxygen gradient, to lay their eggs in the mesenteric vessels (mainly inferior mesenteric vein tributaries and the superior hemorrhoidal vein). The eggs reach the colorectal lumen and are excreted with feces. A similar process usually takes place after infection by* S. haematobium*, with the difference being that the females lay their eggs in the perivesical venous plexus and the eggs are eliminated with urine. Despite being typically associated with the urinary tract,* S. haematobium* has also been associated with the colorectum, with rectal perforation reported in the literature [6].

Eggs that fail to reach the intestinal lumen can become trapped in the submucosal layer of the colon or rectum. The retained eggs can cause an inflammatory reaction responsible for the clinical presentation. Hyperplasia, ulceration, microabscess formation, polyposis, and even carcinogenic transformation in the gut wall can occur [1, 7].

Some authors divide the colonic presentation into acute and chronic schistosomal colitis. The difference between acute and chronic colitis depends on whether* Schistosoma* ova are viable. Acute presentation shows the ova in the submucosa and lamina propria, usually accompanied by eosinophil infiltration. In the chronic phase, calcified eggs are found and the infiltrate is predominantly by lymphocytes and epithelioid cells. In some cases, patients can be completely asymptomatic. Fibroplasia and scarring around the calcified ova can lead to clinically significant strictures. Symptoms are usually nonspecific and include abdominal pain, flatulence, and changes in bowel habits. Chronic diarrhea, rectal bleeding, and colonoscopic findings may mimic inflammatory bowel disease. The development of a mass can be confused with malignancy or complicated diverticulitis.

The association between* Schistosoma* and neoplasia is controversial. There are reports of adenomatous, hamartomatous, and hyperplastic polyps associated with schistosomal eggs [8–10]. The association between* S. japonicum* and colonic cancer has been described; however it is poorly understood. In a study of 1229 rectal biopsies and colectomies in patients with schistosomiasis, Ming-Chai et al. found 37.1% cases of large bowel carcinoma. Their comparison to a control group with colonic cancer not associated with schistosomiasis suggested that diffuse colonic involvement and the >10-year history of colonic symptoms could play a role in the development of a malignancy [11]. Another Chinese study from Liu et al. reported 32 cases of colonic carcinoma in 179 cases of colonic schistosomiasis (17.9%), reinforcing the hypothesis that chronic schistosomiasis may have an etiological role in the genesis of colonic carcinomas [12]. A complex mechanism involving chronic inflammation triggered by the entrapped eggs may be responsible for the carcinogenesis associated with helminthic infections.

Proper diagnostic tools for this infection have been developed. In patients with nonspecific intestinal lesions without a suspected diagnosis of schistosomiasis, endoscopic ultrasound evaluation can be enlightening by showing granulomas caused by the eggs. The ultrasound can also provide information regarding other possible diagnoses. The most common diagnostic method is to confirm the parasitic ova in the stool. Rectal biopsy is an effective diagnostic modality since it provides a method of visualizing the eggs. Other tools include direct assays (demonstration of antigen or DNA) and indirect assays (demonstration of antibody in blood via serology). No further tests were indicated in our patient after the histologic confirmation of the eggs. Species differentiation is usually made by microscopic examination of the eggs and epidemiologic information.

Praziquantel is the drug of choice for treating schistosomiasis since it is effective against all species. The exact mechanism of action is unknown, but the drug is active only against the mature worms and depends on the host immune status. Active acute disease and early chronic lesions are indications for this treatment. Chronic lesions such as calcified granulomas and fibrosis may not respond to this treatment. Since the absence of viable eggs in the stool or urine does not exclude schistosomiasis, patients with calcified eggs who are from endemic areas usually receive treatment following the diagnosis. The therapeutic dose is 40 mg/kg body weight as a single dose for* S. mansoni* and* S. haematobium*. For* S. japonicum* and* S. mekongi*, the recommended dose is 60 mg/kg body weight.

Accordingly, schistosomiasis is still an endemic infection in some parts of Brazil and other tropical regions, causing colorectal lesions with unspecific presentation. Diagnosis can be difficult in unsuspected cases.
